# Strong Teens for Healthy Schools: Protocol for evaluating a youth nutrition, physical activity, and civic engagement protocol

**DOI:** 10.3389/fpubh.2025.1654678

**Published:** 2025-09-17

**Authors:** Alexandra L. MacMillan Uribe, Alisha George, Andrew McNeely, Lucy Xin, Erika Largacha Cevallos, Chad Rethorst, Rebecca A. Seguin Fowler, Jacob Szeszulski

**Affiliations:** ^1^Institute for Advancing Health Through Agriculture, Texas A&M AgriLife Research, Dallas, TX, United States; ^2^Texas College of Osteopathic Medicine, University of North Texas Health Science Center, Fort Worth, TX, United States; ^3^Institute for Advancing Health Through Agriculture, Texas A&M AgriLife Research, College Station, TX, United States

**Keywords:** metabolic syndrome, healthy eating, physical activity, school health, positive youth development, cluster randomized controlled trial, middle school students

## Abstract

Early prevention and treatment of metabolic syndrome (MetS) through healthy eating and physical activity are essential for reducing chronic disease risk among youth. The school food and physical activity environment significantly influences children's health behaviors. Yet, interventions targeting schools' health environments are sparse, and none empower middle school students to be change agents. We describe the process and rationale for evaluating Strong Teens for Healthy Schools (STHS), a multilevel middle school civic engagement program promoting healthier school environments through student-driven projects. We will test the efficacy of STHS for reducing MetS risk and improving positive youth development outcomes among students at middle schools where >40% of students identify as Black/African American or Hispanic/Latino (*n* = 20 schools; *n* = 20–25 students per school) through a pilot cluster randomized controlled trial. Schools randomized to STHS intervention will participate in 16 modules on civic engagement, healthy eating, and physical activity, and schools randomized to the delayed intervention group will receive the curriculum one year later. Student outcomes will be measured at four points: within 1 month of recruitment, at end of intervention, at start of next school year, and at end of next school year. Secondary outcomes include students' perceptions of peers' health behaviors and environmental assessments through photovoice. Evaluating the efficacy of STHS in improving MetS and promoting positive youth development will provide initial evidence on improving physical activity and nutrition outcomes at individual, social, and environmental levels, paving the way for larger-scale studies and informing dissemination efforts.

**Clinical Trial Registry Number:** ClinicalTrials.gov ID NCT05867433.

## Introduction

Metabolic syndrome (MetS) is a cluster of risk factors that increases an individual's risk for cardiovascular disease and type 2 diabetes (1). These risk factors can appear early in childhood, leading to lifelong chronic disease morbidity and mortality (2, 3). A diagnosis of pediatric MetS, following the International Diabetes Federation (IDF) criteria and modified to meet appropriate pediatric thresholds, requires the presence of abdominal obesity plus at least two of the following risk factors: hyperglycemia or insulin resistance, hypertension, elevated triglycerides, or low levels of high-density lipoprotein (4). In the United States, MetS risk factors are increasingly evident earlier in life and affect races and ethnicities disproportionately (5, 6). Among adolescents aged 12 to 19 years, 10.1% have MetS, and 73.2% have at least one condition related to MetS (5). Among overweight adolescents, one in three is affected by MetS (7). Cardiometabolic risk factors, such as obesity and type 2 diabetes, are more prevalent among adolescents who identify as African American/Black and Hispanic/Latino compared to their White counterparts (8).

Early prevention and treatment of MetS, through the promotion of healthy lifestyle behaviors, is essential for reducing the incidence of cardiovascular disease and type 2 diabetes throughout life (9, 10). Diet quality and physical activity are two healthy lifestyle behaviors closely linked to MetS (11). However, among adolescents, 66% exhibit poor diet quality, and only 8% meet the recommended 60 min of physical activity per day, with a smaller proportion of African American/Black and Hispanic/Latino adolescents adhering to dietary and physical activity recommendations (12, 13). Interventions that promote physical activity and healthy eating behaviors are needed to reduce MetS and improve the health of children and adolescents.

In the U.S., >50 million youth spend half of their waking hours and consume half of their meals at school, making it an ideal setting for diet and physical activity interventions (14). Prior studies have shown that changes to the middle school food and physical activity environment have a strong, positive impact on children's dietary and physical activity behaviors and weight outcomes (15–18). As established by ecological systems theory, health-related behaviors occur within a dynamic system where surrounding environments can encourage, discourage, or prohibit behaviors (19). The school food and physical activity environment has a major impact on children's health behaviors and certain MetS conditions, such as adiposity, making it essential for school health environments to promote healthy eating and physical activity to prevent and treat MetS (17, 20).

A growing body of research demonstrates the potential of youth civic engagement activities to catalyze environmental change and create positive health environments, which can also lead to positive youth development outcomes (e.g., competence, confidence, connection, character, and caring) (21). Positive youth development, in turn, promotes prosocial behaviors by engaging youth in their community and can enhance resilience, self-esteem, and academic success (22, 23). Additionally, civic engagement in this developmental stage is positively linked to future income and educational attainment in adulthood—two critical social determinants that influence MetS conditions and lifelong health (22, 24, 25). Despite the many benefits, few youth civic engagement interventions that focus on nutrition or physical activity are documented in the literature, and none have been implemented or rigorously evaluated in a middle school setting (26–28).

As a result, we adapted Strong Teens for Healthy Schools (STHS)—a multilevel middle school civic engagement program that promotes healthier school environments through student-driven projects—from Change Club. Change Club, a 24-week (two times per week) evidence-based civic engagement program, aims to improve health behaviors and outcomes among adults in underserved communities by guiding individuals to create healthy environmental changes within their communities (29). For Change Club, mechanisms of changing physical activity and healthy eating behaviors were focused on social cognitive theory constructs at the individual level and influencing multiple environments within a socioecological framework (e.g., social support). The STHS program provides group-based physical activity and nutrition education to promote healthy behaviors and reduce MetS risk.

The purpose of this manuscript is to describe the process and rationale for developing STHS, as well as the design of pilot cluster randomized controlled trial used to test the efficacy of STHS for: (1) reducing MetS syndrome risk, and (2) improving positive youth development outcomes among middle school student at schools where >40% of the student body identifies as Black/African American or Hispanic/Latino. We chose to recruit schools with a high prevalence of Black/African American or Hispanic/Latino students due to the disparities in cardiometabolic risk factors, coupled with lower engagement in healthy eating and physical activity than their White counterparts, as described above (12, 13). Additionally, we describe processes for assessing the impact of STHS in changing perceived health-related behaviors among the peers of STHS participants (social level) and the school health environment (environmental level).

## Methods and analysis

### Design

**Figure 1:**
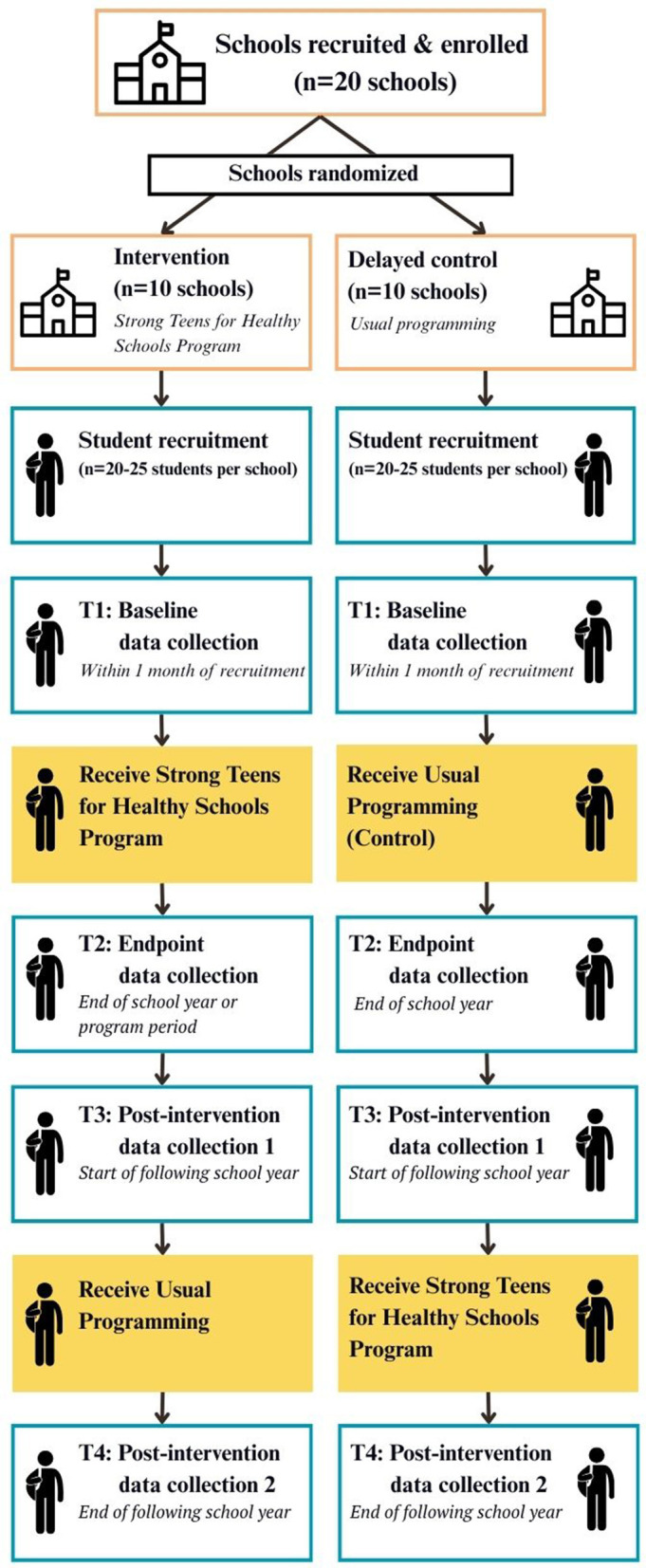
Study design and program implementation for cluster-randomized controlled trial to evaluate the strong teens for healthy schools program..

We will recruit two cohorts of schools (*n* = 20 schools, total) to participate in a 9-month pilot cluster randomized controlled trial. The study design processes—including school recruitment, enrollment, and randomization, as well as student recruitment, data collection timepoint, and program participation—are illustrated in Figure 1 and described in detail below. Once a school agrees to participate, it will be randomized to receive the STHS intervention immediately or a delayed intervention condition (i.e., control group). The delayed intervention group will receive the curriculum to implement at their discretion, one school year after serving as a control. We will collect pre-(beginning of the academic year; T1) and post-test outcomes (end of the academic year; T2) for intervention and delayed intervention conditions. Additionally, for the intervention group, we will gather outcomes at 4 months (T3) and 12 months (T4) post-intervention to assess the short- and long-term maintenance of the intervention effects. The trial has been registered on clinicaltrials.gov (NCT05867433). The Institutional Review Board at Texas A&M University approved all measures, procedures, and protocols (IRB2023-0530D).

#### Study setting

The study will take place in Texas, United States (US). Texas is the second most populous state in the country (30.5 million residents in 2023) and had the highest population increase of any state (473,000 new residents) in 2023 (14). Among Texas residents, 13.6% identify as non-Hispanic Black/African American, 39.8% as Hispanic/Latino, and 76.8% as White (14). Although state-level prevalence of MetS is not available, 21.5% of children 10–17 years old have obesity in Texas, one of the highest prevalences in the nation, stressing the importance of programs addressing MetS in this state (30). Furthermore, obesity rates are nearly twice as high for African American (26%) and Hispanic (29%) children as for Non-Hispanic White children (17%) (31).

The STHS program will be implemented in partnership with Texas A&M AgriLife Extension agents (32). Texas A&M AgriLife Extension is an education agency that provides programs, tools, and resources on a local and statewide level (33). AgriLife Extension has 250 offices (one in almost every Texas county), 900 extension educators, and a network of almost 100,000 volunteers that support the delivery of programs (33–35). Extension agents within the Family and Community Health and 4-H Youth Development units aim to help Texans better their lives through science-based educational programs designed to improve the overall health and wellness of individuals, families, and communities (36, 37). Agents deliver programs in the local communities (e.g., worksites, recreation centers, community events, military bases), as well as within local schools (36, 37).

#### Study eligibility

The inclusion criterion for schools is that >40% of the student body identifies as Black/African American or Hispanic/Latino. With regards to students, all students at a school that is randomized to receive STHS can participate in STHS and are not required to participate in study-related activities. For students who participate in study-related activities, the inclusion criteria are being in the fifth through eighth grades and enrolled at a middle school receiving STHS or the control condition. The exclusion criteria for students who participate in study-related activities are participation in a weight loss program in the past 3 months or the presence of a condition that prevents participation in physical activity.

### Selection of subjects

#### Recruitment

School recruitment will occur using a two-pronged approach. First, we will use the Texas Education Agency website to identify contact information for all middle schools in Texas that meet inclusion criteria (38). We will recruit schools using emails, phone calls, and information folders dropped off at eligible schools in Texas. Simultaneously, we will work with AgriLife Extension Family and Community Health and 4-H agents to identify schools that may be interested in participating in the study. We will hold in-person or virtual meetings with school representatives (e.g., school health advisory councils) to provide an in-depth program description. School administrators from all schools that enroll in the study will sign a memorandum of understanding (MOU) agreeing to participate.

Within schools, we will recruit 5th through 8th grade students (*n* = 20–25 students per school). Eighth-grade students will only be recruited in schools that also have an attached high school where eighth-grade students matriculate, so that they can be assessed at follow-up. Otherwise, we will not recruit 8th-grade students because their transition into high school could introduce new individual and/or environmental factors that may affect study outcomes. Likewise, we will only recruit 5th through 8th grade students from elementary schools if the grade does not matriculate into another school the following year. We will disseminate information for participating in the STHS program via school-wide announcements, flyers posted at the intervention schools, and school-sponsored events. Students and their parents will also receive an email with information about participating in the research study and information on how to contact the research team if they are interested in their student(s) participating. We will also host information sessions for parents and students before and after school, where they can ask more questions about the program. Prior to signing consent forms, the research team will screen participants for inclusion and exclusion criteria. If the student qualifies for the study, the parent will be asked to read and return a consent form. Students will be asked to assent to participate in all study procedures.

#### Randomization

Randomization of schools into the immediate intervention or delayed intervention control conditions will be carried out by a statistician using an automated computer program. Randomization will occur in two blocks of ten schools, at an equal one-to-one ratio. The statistician will keep the resulting group assignments in a downloaded file and restrict file access from the study team. The research team will be blinded to randomization during the recruitment process. Once a school is committed to the study (i.e., signed MOU), the statistician will inform the study team to which group the school has been randomized. Each school will be randomized in the order in which they commit to participating in the study.

#### Delayed intervention control group

The delayed intervention control group will continue with usual care during the first school year, as they will not be asked to add or remove any of their current physical activity, healthy eating, or positive youth development programming. During that year, students will be recruited to take part in data collection within 1 month of the student recruitment period (T1) and at the end of the school year (T2). After T1 and T2 data collection is complete, the control schools will receive the STHS intervention and $3,000, as described below, to implement at their discretion.

### Interventional methods

#### Intervention development and refinement

**Figure 2:**
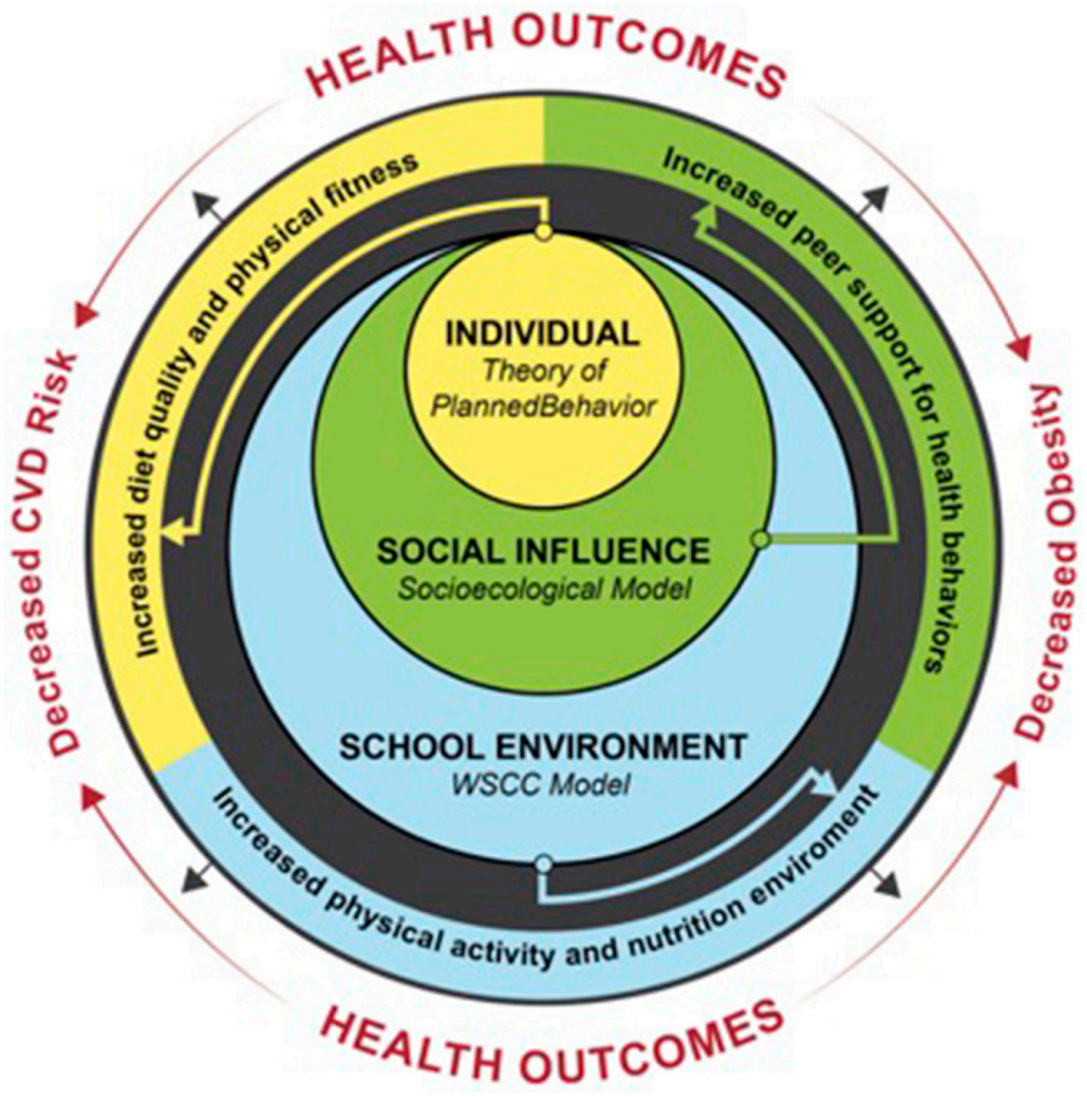
Strong teens for healthy schools-change club theoretical frameworks and expected outcomes.

As previously stated, STHS was developed by adapting Change Club, an evidence-based civic engagement program that aims to improve health behaviors and outcomes among adults in underserved communities by guiding individuals to create healthy environmental changes in their community. In our STHS adaptation, utilizing evidence-based intervention (EBI) mapping (39, 40), we adapted mechanisms of behavior change to utilize the theory of planned behavior as opposed to social cognitive theory, continued to focus on interpersonal social support, and narrowed the focus of environmental change from the community-level to the 10 domains of the Whole School, Whole Community, Whole Child (WSCC) model (Figure 2). Finally, we adapted the program to allow greater flexibility in delivery, considering the variability of instructional schedules across schools [i.e., structured 24-week program to 16 flexible 1-h modules (or 32 thirty-minute sessions)].

Additional refinements were made to STHS by conducting preliminary qualitative interviews with intended program implementers—AgriLife Extension agents (*n* = 20) and middle school staff (*n* = 12) from Texas. Implementers provided feedback on the strengths and weaknesses of STHS's components (i.e., curriculum, resources, and training). This study is published elsewhere (41). Briefly, findings from this study emphasized the following: potential challenges due to variations in students' reading levels and understanding of key concepts, the need for implementer training and support to facilitate consistent delivery of the core curriculum components, adding alternative activities that could overcome school resource- and scheduling-related challenges, and the importance of activities and resources to actively engage students (41). These findings informed additional modifications made to refine STHS (Table 1).

**Table 1:** Strong teens for healthy schools curriculum refinement from preliminary efforts.

**Issue**	**Change made to STHS**	**Data sources/reason**
Curriculum comprehension	Lowered the reading level, simplified key concepts and added examples after a concept is introduced (e.g., provided examples of assets like buildings and people).	In the **preliminary qualitative work** and **pilot testing**, extension agents and teachers discussed students' challenges with the reading level and lack of understanding of key concepts.
	Adding class engagement activities (e.g., thumbs up/down) to assess students' existing knowledge or their takeaways from curriculum topics.	In the **preliminary qualitative work**, extension agents and teachers emphasized the importance of student engagement, and in our **pilot testing**, research staff observed that students were not as engaged as we had hoped with the curriculum.
Curriculum structure	All lessons were structured as follows – overview, description of the topic with a “say” statement that emphasizes key points and provides additional talking points, activity, discussion questions, and reminders.	In our **pilot testing**, research staff observed inconsistency in session structure.
	Optional technology free activities—All sessions that required the use of technology were supplemented with an optional activity.	In our **preliminary qualitative work**, extension agents and teachers suggested a need for activities to fill additional time or replace activities that required technology. In the **pilot testing**, the teacher said some of the websites in the curriculum were blocked and that they didn't know how to use the classroom smart board until the end of year.
Additional resources	Complementary student workbooks were created for students to document and track progress on their project.	In the **pilot testing**, research staff observed that teachers had a hard time keeping track of important handouts related to the civic engagement project.
	The implementation guide's appendix was updated with resources, such as supporting videos to serve as a visual aid for key concepts (e.g., heart disease, mindfulness).	In the **pilot testing**, research staff observed that some of the videos in the curriculum were no longer available online.
Training	Research staff provide structured and comprehensive training	In our **preliminary qualitative work**, extension agents wanted more resources on how to work with students, and school staff discussed the importance of training to facilitate implementation. In the **pilot testing**, research staff observed a need for more structured training for teachers that could also be replicated if the STHS implementer switches during the year.
	Added a student implementer program where students at local colleges provide support to teach sessions. Student serves whatever role is needed as the lead educator deems appropriate (e.g., team-based teaching or classroom management support).	In the **preliminary qualitative work**, extension agents and school staff groups wanted a dedicated person to implement program. During **pilot testing**, the interviewed teacher wanted more support in implementing the session, and the research team ended up serving as additional support when they attended the observations. Additionally, the research team noticed a need for continuity when an implementer wasn't available or switched during the program.

STHS, Strong Teens for Healthy Schools.

We also pilot-tested STHS at one school (*n* = 5 students) as part of an after-school program. During pilot testing, the research team observed five STHS sessions, taking observational notes and having informal discussions with the teacher implementing the program. The teacher also participated in a structured interview, providing feedback about their experience delivering STHS. The research team's observations and informal discussions with the teacher revealed issues with student engagement, challenges with technology-based activities, inconsistency in session structure, difficulties with keeping track of important handouts related to the civic engagement project, and the need to create a contingency plan when an implementer was unavailable or left during the program. Like the implementer interviews described above, both research team observations and the teacher interview revealed potential challenges due to variations in students' reading levels and understanding of key concepts, as well as the need for implementer training and support to facilitate the consistent delivery of core curriculum components. These findings also informed additional modifications made to refine STHS (Table 1).

#### Intervention content

For this study, STHS includes 16 one-hour modules, which could also be delivered as 32 thirty-minute sessions to provide flexibility, that provide education and activities on civic engagement, healthy eating, and physical activity (Table 2). Additionally, students will set healthy eating and physical activity goals using the Specific, Measurable, Achievable, Relevant, and Time-bound (SMART) goal approach and receive support for maintaining individual-level healthy eating and physical activity behaviors. They will also identify, plan, and implement a school health environmental change project. Each STHS school will receive a $3,000 stipend to support the health environmental change project(s).

**Table 2:** Overview of strong teens for healthy schools curriculum topics.

**Module**	**Type**	**Session tile (30 min)**	**Activities**
1A	CE	Introduction & fostering engagement	1.1 Program overview 1.2 Strong teens bingo (i.e., icebreaker) 1.3 Setting club rules 1.4 Activity on the types of environmental change
1B	PA	Importance of physical activity	1.5 Types of physical activity discussion 1.6 SMART goal setting for physical activity 1.7 Fitness charades (i.e., active game) 1.8 Extended activity (i.e., try a new type of physical activity at home)
2A	CE	Team building	2.1 Cup stacking challenge (i.e., team building) 2.2 Advocacy gallery walk (i.e., activity to identify personal interests)
2B	HE	Healthy eating basics	2.3 Kahoot about healthy eating 2.4 SMART goal setting for healthy eating 2.5 Extended activity—download MyPlate Plan App to use at home
3A	CE	PhotoVoice projects	3.1 Photovoice part I (i.e., students complete an audit of their school health environment) 3.2 Small group discussion on photos taken
3B	PA	Facilitators & barriers to physical activity	3.3 Physical activity SMART goal check-in 3.4 Step awareness (i.e., activity that discusses how the environment affects physical activity). 3.5 Facilitators & barriers relay race (i.e., activity that discusses barriers and facilitators for physical activity)
4A	CE	Choosing a strategy & advocacy skills	4.1 Examples of environmental change projects 4.2 Create school change vision board
4B	HE	Supports & challenges to healthy eating	4.3 Healthy eating SMART goal check-in 4.4 Stand up sit down (i.e., activity to assess sugar knowledge) 4.5 Nutrition facts label—sugar 4.6 Grab & Go (i.e., activity that discusses barriers and facilitators for healthy eating)
5A	CE	Identifying stakeholders	5.1 Identifying stakeholders for environmental change 5.2 Contacting stakeholders for environmental change
5B	PE	Types of fitness	5.3 Physical activity SMART goal check-in 5.4 Fitness rotation (i.e., activity to discuss three types of fitness—aerobic, muscular strength/endurance, flexibility)
6A	CE	Asset mapping	6.1 School asset mapping 6.2 Personal asset mapping 6.3 Stakeholder check-in
6B	HE	Fruits, vegetables, & whole grains	6.4 Healthy eating SMART goal check-in 6.5 Mystery bucket (i.e., matching foods to their MyPlate category) 6.6 Nutrition facts label—dietary fiber
7A	CE	Leadership development	7.1 Leadership qualities discussion 7.2 Sneak-A-Peak activity (i.e., activity that focuses on problem solving and communication) 7.3 Designating group roles 7.4 Stakeholder check-in
7B	PA	Heart health	7.5 Physical activity SMART goal check-in 7.6 Heart health exploration (i.e., discuss how physical activity affects the heart) 7.7 Mindful walking
8A	CE	Vision planning	8.1 Develop mission statement for project 8.2 Create logic model for project
8B	HE	Proteins, fats, & dairy	8.3 Healthy eating SMART goal check-in 8.4 Nutrition facts label—fats 8.5 Healthy eating jeopardy 8.6 Optional activity—the ingredient game (i.e., create healthy meals using random recipe cards)
9A	CE	Action planning	9.1 Would You rather activity (i.e., activity for students to think about personal preferences for physical activity and healthy eating). 9.2 Develop action plan for project
9B	CE	Evaluation & monitoring	9.3 The blind polygon (i.e., game to teamwork, goal alignment, and organization skills) 9.4 Develop evaluation plan for project
10A	CE	Resources & support	10.1 Evaluate resources needed project 10.2 Project check-in #1 10.3 Optional activity—Papa's Sushiria Online Game (i.e., time management game)
10B	PA	Physical activity & SMART goals	10.4 Physical activity SMART goal check-in 10.5 Race for the truth (i.e., activity to visual social norms around physical activity).
11A	CE	Leadership development	11.1 Project check-in #2 11.2 Leaders You admire activity (i.e., activity to discuss leadership characteristics) 11.3 Silent leadership game (i.e., activity to discuss leadership characteristics) 11.4 Extended activity—community bingo (i.e., meet local leaders)
11B	HE	Healthy eating & SMART Goals	11.5 Healthy eating SMART goal check-in 11.6 Whyville snack shack games (i.e., game to read nutrition label) 11.7 Optional activity—food charades
12A	CE	Project implementation	12.1 Time squared (i.e., time management activity) 12.2 Project work time 12.3 Extended activity—community bingo check-in
12B	HE	Physical activity & SMART goals	12.4 Physical activity SMART goal check-in 12.5 Physical activity Cube (i.e., game to learn about active breaks) 12.6 Optional activity—quick thinks (i.e., review different types of physical activity)
13A	CE	Project implementation	13.1 Paper airplane (i.e., leadership and communication game) 13.2 Project work time
13B	PA	Healthy eating & SMART goals	13.3 Healthy eating SMART goal check-in 13.4 Breakfast around the world (i.e., MyPlate education about breakfast in different countries) 13.5 Optional activity—Food Knockout (i.e., game to review healthy foods)
14A	CE	Resources	14.1 Desert island game (i.e., game to learn about prioritization) 14.2 Project work time 14.3 Extended activity—Cake Mania Online Game (i.e., online time management game)
14B	CE	Project implementation	14.4 Colored pieces (i.e., game to learn about prioritization) 14.5 Project work time
15A	CE	PhotoVoice projects	15.1 Photovoice part II (i.e., students complete an audit of their school health environment and discuss changes over the year) 15.2 Small group discussion on photos taken
15B	CE	Project implementation	15.3 Final project work time 15.4 Optional Activity—find the Ace of Spades (i.e., time management and planning game) 15.5 Extended activity—final community bingo—check-in
16A	CE	Final SMART goal check-ins project reflections	16.1 Final SMART goal check-in 16. 2 Reflection snowballs (i.e., game to reflect on project) 16.3 Reflection discussion
16B	ALL	Project presentations awards ceremony	16.4 Project presentation 16.5 Awards ceremony

Extended activities are things that youth can do at home. Optional activities can replace the main activities in the curriculum. CE, civic engagement; HE, healthy eating; PA, physical activity.

#### Intervention theoretical framework

The STHS program is based on the Social Ecological Model (SEM), a multifaceted framework for understanding the effects of personal, social, and environmental factors on behaviors (42). The SEM posits that individual behavior is shaped by multiple levels of influence (42), including individual, social, and environmental levels, which are common targets within healthy eating and physical activity interventions (43). At the individual level, STHS incorporates the theory of planned behavior (TPB) to promote healthy eating and physical activity. The TPB posits that strong behavioral intent increases the likelihood of an individual engaging in health behaviors (44), and behavioral intent is influenced by behavioral beliefs (e.g., knowledge and attitudes), social and subjective norms, and perceived behavioral control (44). Group discussions and interactive activities focus on improving behavioral beliefs, self-monitoring, and goal setting to increase perceived behavioral control, whereas group-based activities are utilized to change subjective norms about how peers engage in healthy eating and physical activity. On a social level, STHS activities emphasize civic engagement, including cultivating support for fellow students participating in physical activity and nutrition, establishing a shared purpose, mission, and vision for STHS, and building advocacy skills to promote positive changes in school physical activity and healthy eating environments. Finally, at the school level, students identify a beneficial change they can make to their school nutrition or physical activity environment and develop, implement, and evaluate that change.

#### Intervention implementation

The STHS program can be implemented before, during, or after school and is designed to be delivered by Extension Agents (Family and Community Health or 4H) or teachers. Once a school decides to participate, they will select a primary implementation lead—the school staff member or Extension Agent—and discuss who will provide additional implementation support, including the research team who will be available to substitute for the lead implementer, as needed. For the primary implementation lead(s), the research team will provide 1 hour of training, either online or in person. The training includes a curriculum overview, as well as information on additional resources (e.g., student workbook, classroom management resources), how to complete the fidelity checklist, student projects, and funding. The implementer will also receive a tote including all the small supplies they might need to deliver the curriculum (e.g., cones, cards, markers). Additional implementation support following the training will be provided, as needed, by the research team.

Additionally, for this study, the research team will recruit and train undergraduate university students to support curriculum delivery at intervention sites and ensure fidelity checklists are completed. Since STHS is being implemented across the state, student implementers are recruited from local colleges and universities as peer mentors who are closer in age to STHS student participants. Student implementers receive the same basic training provided to the implementation lead (described above); however, they also attend weekly check-in meetings with the research team. Meetings serve as a learning collaborative where all peer mentors discuss their successes and challenges with STHS implementation, provide updates on where they are in the implementation process, and provide feedback and support to one another. Additionally, student implementers participate in short trainings and discussions on various topics, including SMART goals, positive youth development, mentorship styles, etc. Finally, student implementers are reminded to work with their implementation leads to complete the fidelity checklists for each session that is complete. In the event that student implementers leave, a contingency plan has been developed to minimize interruptions or inconsistencies in curriculum delivery. We anticipate any interruption to be minimal since the students are not the primary implementers of the curriculum. Furthermore, our research team is fully trained in delivering the curriculum and will be able to step in to support curriculum delivery while another student is identified, hired, and trained.

### Measures

All student participant outcomes will be measured for student participants at both the intervention and delayed intervention control group schools within 1 month of the student recruitment period (T1), at the end of the school year or intervention period (T2), at the start of the next school year (T3), and at the end of the next school year (T4; Figure 1). Data collection sessions are estimated to take 45 minutes per student, and measures will be administered to students as a group. The two primary individual-level outcomes for this study are changes in MetS and positive youth development. Secondary individual-level outcomes include: (1) individual metabolic risk factors (blood pressure, fasting blood glucose, waist circumference, HDL cholesterol, and triglycerides); (2) body mass index percentile; (3) fruit and vegetable consumption; (4) accelerometer-derived physical activity, sedentary time, and sleep; and (5) cardiorespiratory fitness. Finally, other outcomes we will survey include TPB constructs related to nutrition and physical activity, nutrition- and physical activity-related behaviors, and youth's engagement with the school health environment. At the social level, we will assess students' perceptions of their peers' physical activity and healthy eating. At the environmental level, we will: (1) measure students' perceptions of the school health environment; (2) have school administration and staff complete a school environment assessment; and (3) conduct photovoice sessions. Student participants in both the intervention and the delayed intervention control group will receive a $50 gift card for each data collection session they complete (T1 to T4; $200 maximum for completing all sessions).

#### Individual-level outcome measures

##### Metabolic syndrome

Participants' MetS will be categorized dichotomously (yes or no) using the IDF definition of MetS for children and adolescents aged 10 to < 16 years old—abdominal obesity plus at least two of the four other MetS risk factors. The IDF uses the following criteria to define risk for each component: (1) waist circumference >90th percentile (i.e., abdominal obesity); (2) systolic blood pressure >130 or diastolic blood pressure >85 mmHg; (3) triglycerides >150 mg/dl; (4) high-density lipoprotein cholesterol (HDL-C) < 40 mg/dl; and (5) fasting blood glucose >100 mg/dl (45). Each risk factor will also be analyzed individually as a continuous variable as a secondary outcome.

Waist circumference will be measured using a medical-grade tape measure with Gulick spring attachment, and it will be measured at the midpoint between the floating rib and iliac crest. The measure will be performed three times and an average of the two closest measures will be used. Blood pressure will be measured using an automated Omron sphygmomanometer and an appropriately sized cuff on the children's upper right arm. As needed, participants will be asked to remove or pull up any bulky garments covering their upper right arm before testing begins. Participants will be asked to remain seated for 5 min before the measurement is taken. Blood pressure will be measured three times, and the two closest measurements will be averaged. For glucose, HDL-C, and triglycerides, participants will be directed to complete an overnight fast. A single capillary blood sample will be collected and inserted into a portable CardioChek Plus analyzer to measure triglycerides, HDL-C, and glucose. Only one measurement will be collected.

##### Positive youth development

We will measure positive youth development using the 5 C's Model of Positive Youth Development Scale-Short Form (PYD-SF) (46). The PYD-SF is a valid and reliable 34-item scale for youth 10–18 years old that assesses the strength of psychological, behavioral, and social development in youth. The five dimensions measured are (1) competence (sense of proficiency); (2) confidence (self-worth, self-efficacy); (3) character (adherence to societal and cultural rules); (4) connection (bonds with people and institutions); and (5) caring (sympathy and empathy toward others). Each positive youth development dimension will be calculated as a mean score, and the average across all dimensions will serve as the primary outcome.

##### Body mass index percentile

BMI percentile will be calculated using the standard formula and plotted on the Centers for Disease Control and Prevention (CDC) BMI-for-age clinical charts for the participants' corresponding sex. Height will be measured three times in succession to the nearest 1/8 inch using a portable SECA stadiometer (Seca Corporation, Hamburg, Germany). An average of the two closest measures will be used. Weight will be measured three times in succession to the nearest 0.1 pound using a portable Tanita body composition scale (Tanita Corporation, Tokyo, Japan). An average of the two closest measures will be used.

##### Fruit and vegetable consumption

We will use subdermal carotenoid levels as a proxy for fruit and vegetable intake. We will measure subdermal carotenoid levels using the Veggie Meter machine (Longevity Link Corporation, Salt Lake City, Utah, United States). The Veggie Meter is a non-invasive, portable machine that measures subdermal carotenoid levels using resonance Raman spectroscopy. The Veggie Meter provides an objective assessment of fruit and vegetable intake through a direct measure of dietary carotenoids and has been successfully used in school settings (47). Prior to taking the measure, participants will be asked to wash their hands or use an alcohol wipe to clean their non-dominant ring finger. The participant's non-dominant ring finger will be placed into the Veggie Meter finger slot, and the measure will be taken three times in succession. An average of the three measurements will be used.

##### Physical activity, sedentary time, and sleep

Physical activity, sedentary time, and sleep will be assessed using a triaxial Actigraph GT3x+ accelerometer (ActiGraph Corporation, Pensacola, Florida, USA). Participants will be instructed to wear the accelerometer on their non-dominant wrist for 24 h on seven consecutive days, and participants will only be asked to remove them for showers or activities where the devices could be submerged in water (e.g., swimming, bathing). Accelerometer non-wear time based on triaxial data was assessed using a validated algorithm (48). Participants who provide data for at least 16 h/day over a minimum of 3 weekdays and 1 weekend will be included in the analysis.

Accelerometer raw data will be collected at a frequency of 30 Hz, calibrated to local gravity, and expressed as Euclidean norm minus one using 5-s epochs. Time gaps identified as non-wear time through each 24-h interval that reached the validation criteria (≥16 h) will be imputed at the raw-data level based on average values across days. All valid days will be normalized to 24 h. Each epoch in a 24-h window will be classified as sedentary behavior (< 35.6 mg), light physical activity (≥35.6 and < 201.4 mg) or moderate-to-vigorous physical activity (≥201.4 mg) using the Hildebrand cut-off points recommended for children (49–51). Sleep time will be obtained using the Heuristic algorithm that examines the distribution of change in Z-angle. This algorithm differentiates sleep from other inactivity windows by calculating the longest sustained period of inactivity with the lowest number of interruptions in a 24-h time window (52). Time spent in moderate-to-vigorous physical activity, light physical activity, sedentary behavior, and sleep will be weighted according to weekdays and weekends (5:2 ratio) and averaged across days for each participant using a midnight-to-midnight day definition. The accelerometer data will be processed using the “GGIR” package version 2.9.1 in R software, version 4.3.0 (R Foundation for Statistical Computing, Vienna, Austria) (53).

##### Cardiorespiratory fitness

We will use the FitnessGram PACER multistage aerobic capacity test to assess cardiorespiratory fitness (VO2 max). After observing a demonstration, children will complete the test in groups of five or six. Children will run back and forth 20 m, with an initial running speed of 8.5 km/h and a progressive 0.5-km/h increase in running speed every minute. A research team member will lead the children through the test to provide instructions and pacing. As the test continues, it becomes progressively harder. The number of laps completed will be put into the standard prediction equations with their sex and age to estimate VO_2_ max.

##### Other measures

On the student survey, we will assess TPB psychosocial constructs—attitudes, subjective norms, perceived behavioral control—related to physical activity and healthy eating (54–56). We will also evaluate time spent being active at school, outside of school, and time spent being sedentary outside of school using the Youth Activity Profile (57). Related to healthy eating, students will complete the Texas School Physical Activity and Nutrition (SPAN) food frequency questionnaire that asks about how frequently they consume 20 different foods and ten drink categories. Responses will then be used to calculate the SPAN Healthy Eating Index and its subscales (healthy and unhealthy classifications) (58, 59). Finally, we will evaluate students' engagement with the school health environment using the Youth Engagement and Action for Health (YEAH!) survey (60). At baseline, sociodemographic variables will be collected and include questions on students' date of birth, sex (male, female, or not listed with the option to provide fill in their response), grade, Hispanic ethnicity (yes or no), race (American Indian, Asian, Black or African American, Native Hawaiian or Pacific Islander, White, or Other with the option to fill in their response).

#### Social level outcomes

##### Perception of peers physical activity and healthy eating

To assess perception of peers' physical activity, we will use a questionnaire developed as part of the Trial of Activity for Adolescent Girls (TAAG) (61). The questionnaire asks participants to identify their three closest friends and, for each friend, answer six items about their experiences engaging in physical activity with the friend. A second questionnaire, based on the TAAG questionnaire, was developed to assess the perception of peers' healthy eating behaviors. For both surveys, five items are yes/no response variables, and each item's score reflects the number of friends for which the item is answered in the affirmative. Additionally, one item assesses the frequency of physical activity with each friend with a 5-point ordinal scale ranging from never to five or more times per week. A total score will be calculated across all items for each behavior, with higher scores representing higher levels of perceived peer engagement in physical activity or healthy eating.

#### Environmental level outcomes

##### Participant perception of school health environment

We will assess students' Perceptions of the Environment and Patterns of Diet at School (PEA-PODS) using a validated survey (62). The PEA-PODS survey is 40 items scored on 5-point Likert scales, and it includes questions about students' perceptions of physical activity and nutrition policies and practices (11 items), teacher wellness policies and practice (16 items), cafeteria policies and practice (7 items), and recess policies and practices (6 items). Each subscale will be scored individually, and items will be aggregated across constructs to create an overall score (higher scores related to a better perceived environment).

##### School environment assessment

School staff will provide an assessment of the school health environment by completing a School Physical Activity and Nutrition Environment Questionnaire (63). The questionnaire includes three parts—administration, physical education, and food service—and each part will be filled out by the most knowledgeable person on each topic. However, if a school staff member serves in two of the previously mentioned positions, they may fill out the survey once for both positions. For the administration portion of the survey, 24 questions ask about school health advisory councils, parent-teacher organizations, school-wide participation in physical activity or nutrition initiatives, sharing of health information with parents, as well as general food and nutrition policies for the school. For the physical education portion of the survey, 33 questions evaluate the frequency and content of physical education, active commuting to school programs or opportunities, availability of physical activity facilities and policies, fitness assessments and reporting, and organized sports and physically active clubs. Finally, the food services portion of the survey uses 20 questions to assess policies and practices at breakfast and lunch, participation in federal meal programs, and foods that are available on campus throughout the school day.

##### Photovoice

We will qualitatively measure changes in students' perceptions of the school food and physical activity environment using a photovoice approach. Photovoice is a participatory research method where participants use photography and storytelling to document and reflect on their experiences (64), which we have embedded as an activity in the STHS curriculum as part of STHS module three (initial assessment) and module 15 (reassessment). For module three, participants will also identify an environmental change to make as part of their STHS project, and for module 15, they will reflect on environmental changes that have occurred in their school over the course of the program. All students will be anonymized and participate in the photovoice data collection sessions, regardless of consent/assent to participate in the STHS study.

To conduct the photovoice session, we will divide students into 3–5 students per group and instruct each group to assess the food environment or the physical activity environment, with at least one group per school assessing each of the two environments. Each photovoice session will consist of a walkabout to take pictures of the school environment (15–20 min) and the selection of one photo per group to answer questions during an implementer-moderated class discussion (15–20 min). For the walkabout, we will provide each group with an instant print digital camera and instruct participants to build consensus on which features of the school food and physical activity environment to capture. Participants will also have a worksheet to complete after the walkabout, instructing them to note supportive or challenging features in their food or physical activity school environment. Following the walkabout, participants will reconvene in the classroom and use a worksheet with guiding questions, build consensus within their group on selecting one picture to share as part of the moderated discussion with all groups. The worksheet questions are informed by the SHOWeD process, an established methodological approach to photovoice previously used in middle school settings (65–68). The SHOWeD questions facilitate contextualization and exploration of root causes surrounding the photographic image captured by participants. The implementer will then facilitate a class discussion using the same SHOWeD process questions to generate a collaborative discussion.

We will audio-record and take structured observations on all phases of the process (walkabout, picture selection, and discussion). Additionally, we will debrief as a team following each photovoice session, noting the degree of group engagement and consensus-building that occurred during the session, and summarizing the prevalent discussion points among participants.

### Data analysis

#### Sample size

As abdominal obesity is a necessary component of MetS, we powered this study on between-group changes in obesity. Based on the existing literature, we set an alpha of 0.05 and estimated a within-school intraclass correlation coefficient of 0.025 to account for cluster effect, an effect size of *d* = 0.27, and an effect size variability of 0.07. As a result, we will need to recruit 20 participants per school to achieve 80% power to detect a statistically significant change in abdominal obesity between the intervention and control group participants. Previous school- and community-based studies have shown similar effect sizes for changes in obesity-related outcomes (69–72). We will aim to recruit 25 students per school to ensure that we have 20 students available for measures, given that the normal retention rate is 80% in community-based research. We anticipate low participant attrition given that the school setting provides a controlled environment where consistent attendance is expected and buy-in from school administration and teachers often leads to higher student engagement and accountability (73). Furthermore, we will be working with Extension agents who often have strong community ties, fostering trust and rapport with students and families (74).

#### Quantitative analysis

We will use univariate and bivariate statistics to determine the distribution of outcome measures and to identify relevant covariates (75). To test between-group differences in post-intervention MetS and positive youth development, intervention and delayed intervention control group districts will be evaluated using a generalized linear model framework, in which the treatment group (intervention vs. delayed intervention control group) will be the primary independent variable, baseline score as a covariate, post-intervention score is the outcome, and district is a clustering variable (76). Other covariates (e.g., student sex), identified in the preliminary bivariate analyses, will also be included in the model. If missing data occurs, we will mitigate potential biases and loss of statistical power by analyzing multiple imputed datasets under an intention-to-treat approach (77). These analyses will be compared to per-protocol analyses to allow for the assessment of intervention effects under different conditions (78).

#### Qualitative analysis

We will use a thematic approach, informed by the SHOWeD framework, to analyze photovoice data (64, 79). First, audio recordings of group discussions and structured observation notes from each phase (walkabout, picture selection, and discussion) will be transcribed verbatim and uploaded into qualitative data management software for coding. A team of trained researchers will independently review transcripts and observation rubrics, applying an inductive-deductive coding strategy. Deductive codes will be informed by the SHOWeD framework and the study's research objectives, while inductive coding will allow for emergent themes that reflect participants' unique perspectives on environmental challenges and facilitators. To ensure reliability, two researchers will code a subset of transcripts and reconcile discrepancies through discussion, refining the codebook iteratively. A comparative analysis will be conducted between photovoice session three and session 15 to examine changes in student-identified environmental barriers, facilitators, and perceived improvements over time. Key themes and representative quotes will be synthesized to capture students' perceptions of their school environment, and findings will be triangulated with observational data to provide a comprehensive understanding of how students perceive and experience environmental change throughout the program.

## Discussion

This study will evaluate the efficacy of STHS in improving MetS and positive youth development among middle school students. Findings from this study will provide initial evidence regarding the effectiveness of STHS within schools with a high proportion of Black and Hispanic students. If successful at improving outcomes, findings from this study can inform efforts to scale up this program and disseminate it to middle schools throughout Texas, as well as other states. As previously described, there is strong evidence to support the long-term behavioral and socioeconomic benefits of school-based health and civic engagement programs (17, 20–25, 80–85). However, the development and evaluation of STHS will be one of the first initiatives to use civic engagement processes to improve nutrition and physical activity environments within middle schools. Accordingly, this study will shed light on how youth civic engagement programs can be leveraged to influence critical health outcomes, like MetS and diet- and physical activity-related behaviors, as well as contributing to positive youth development.

As an innovation, this study evaluates a middle-school health promotion program that emphasizes student-driven initiatives to make positive changes to students' social and school environments. Previous studies indicate that programs that allow for more autonomy among middle school students in choosing projects or curriculum can foster motivation and engagement and lead to improved developmental outcomes, like desirable social-emotional skills or resilience (86–88). This is critically important, as students' motivation and engagement are major concerns among educators, and engagement and motivation are associated with lower academic achievement and increased absenteeism (89, 90). Furthermore, middle school is an important time when peer-to-peer social processes exert a stronger influence on students' behaviors. By attempting to change social norms around physical activity and healthy eating, STHS has the potential to create healthier peer-to-peer interactions that can positively affect behavioral and health outcomes.

Another unique aspect of this study is the inclusion of photovoice for STHS participants to assess and reassess their school food and physical activity environment. Photovoice has been used extensively with middle school students as an effective method for increasing empowerment and participation; however, it has seldom been used to assess environmental change or enact community-driven strategic action within schools (91, 92). Rather, most previous studies have only used photovoice to capture and characterize photographic images at one point in time, and reviews have identified the enactment of social change as a major challenge to the photovoice approach (91, 92). Utilizing a comparative analysis to assess pre- to post-intervention changes in the perceived food and physical activity environment within schools has strong potential as a robust environmental assessment to assess a community-driven action approach, which could be applied to future studies.

Finally, STHS was specifically designed with program implementation in mind. First, the program can be delivered in 30-min or 1-h sessions to accommodate a variety of before, during, and after-school programming schedules. Additional activities are also provided for each lesson to extend the program length as needed. This allows for better integration into a variety of timetables and implementer experience levels, which were identified as crucial factors in our pre-intervention interviews with middle school staff and Extension agents. We also aligned each lesson's learning objectives with Texas Essential Knowledge and Skills (TEKS), state standards for student knowledge and instruction set forth by the Texas Education Agency (93). Aligning STHS learning objectives with TEKS standards helps educators identify and meet the required TEKS for each grade level and promotes adoption of the curriculum during the school day. Overall, conducting pre-implementation interviews to preemptively address some of STHS's barriers to implementation before the randomized controlled trial allowed us to modify implementation strategies and develop new implementation strategies (e.g., student implementers who meet as part of a learning collaborative) to meet STHS's program delivery needs. We will continue to evaluate these strategies throughout the study.

This study is not without its limitations. First, the STHS program will be delivered in both public and private middle schools in a variety of settings with different policies and administrators. We have hired student implementers to minimize variation and measure curriculum fidelity, allowing us to identify significant deviations. The flexibility in delivering the curriculum, as discussed above, may introduce variability but only affects the frequency of delivery, not the learning objectives of each session. Second, the randomized design with a delayed intervention control group may present challenges for Extension agents or school staff. For example, if school staff planned to deliver STHS during school hours, but they were randomized to receive the delayed intervention control condition, they may seek to replace STHS with a similar program. Given the STHS program's unique approach, it is unlikely that a comparable program is available (i.e., a program that combines civic engagement with nutrition and physical activity education); however, they may choose a program that addresses one of the primary outcomes (e.g., positive youth development). Although we considered traditional non-contract control, school staff and Extension agents expressed ethical concerns with denying programming to community members. Therefore, the delayed intervention control condition was deemed the most appropriate.

Overall, this study will evaluate the efficacy of STHS in improving MetS and promoting positive youth development among middle school students. The findings will also provide evidence on whether STHS is effective in improving physical activity and nutrition outcomes at the individual, social, and environmental levels. If found to be effective, this will pave the way for larger-scale studies and inform efforts to disseminate STHS. The study addresses a critical gap in interventions for middle school students by concurrently emphasizing youth civic engagement, nutrition, and physical activity. By promoting student-driven initiatives and employing innovative methods like photovoice, the STHS program represents a significant advancement in health promotion for middle school students.
